# A novel approach to airway management in Pierre Robin syndrome—a case report

**DOI:** 10.1093/omcr/omac132

**Published:** 2022-12-23

**Authors:** Ajit Bhardwaj, Ritu Grewal, Shaleen Trivedi, Shivinder Singh

**Affiliations:** Dept Of Anaesthesia, & Critical Care, Command Hospital Lucknow, India; Dept Of Anaesthesia, & Critical Care, Command Hospital Lucknow, India; Dept Of Anaesthesia, & Critical Care, Command Hospital Lucknow, India; Dept Of Anaesthesia, & Critical Care, Command Hospital Lucknow, India

## Abstract

Pierre Robin syndrome (PRS) neonates are one of the most difficult cases to intubate even for an experienced paediatric anaesthesiologist. We describe a case of a PRS-related anatomical anomaly that hindered attempts to manage the airway and the final approach that made it possible to insert an endotracheal tube (ETT). We describe the novel use of a video ureteroscope (Olympus URF-V2) as an airway endoscope. A 7-day-old, 2-kg boy was referred to our tertiary care hospital with diagnosed PRS. He was planned for correction of the mandible with mandibular distraction osteogenesis under general anaesthesia. Fibreoptic scope (Olympus, Japan) revealed the epiglottis lying on the posterior pharynx, which could not be manoeuvred. Due to repeated attempts, the patient developed laryngospasm, and his pulse arterial oxygen saturation (SpO2) was reduced to 70%. Following jaw thrust and slight pulling of the tongue with Magill’s Forceps, a 150-cm long and 0.035-inch diameter atraumatic, Roadrunner® hydrophilic polyurethane-coated guidewire was introduced through the working channel of the video ureteroscope into the trachea under the vision (and a 3.5-mm ID ETT was railroaded over it and a definitive airway was established). A flexible fibreoptic ureteroscope may be useful in the management of a difficult airway and may become an important tool in the armoury of an anaesthesiologist. At our institute, which is a tertiary care centre, we are now training and utilising video-ureteroscope as an airway endoscope. To our knowledge, there is no documentary evidence of the use of a video ureteroscope for difficult airway management of a neonate.

## INTRODUCTION

**Figure 1:**
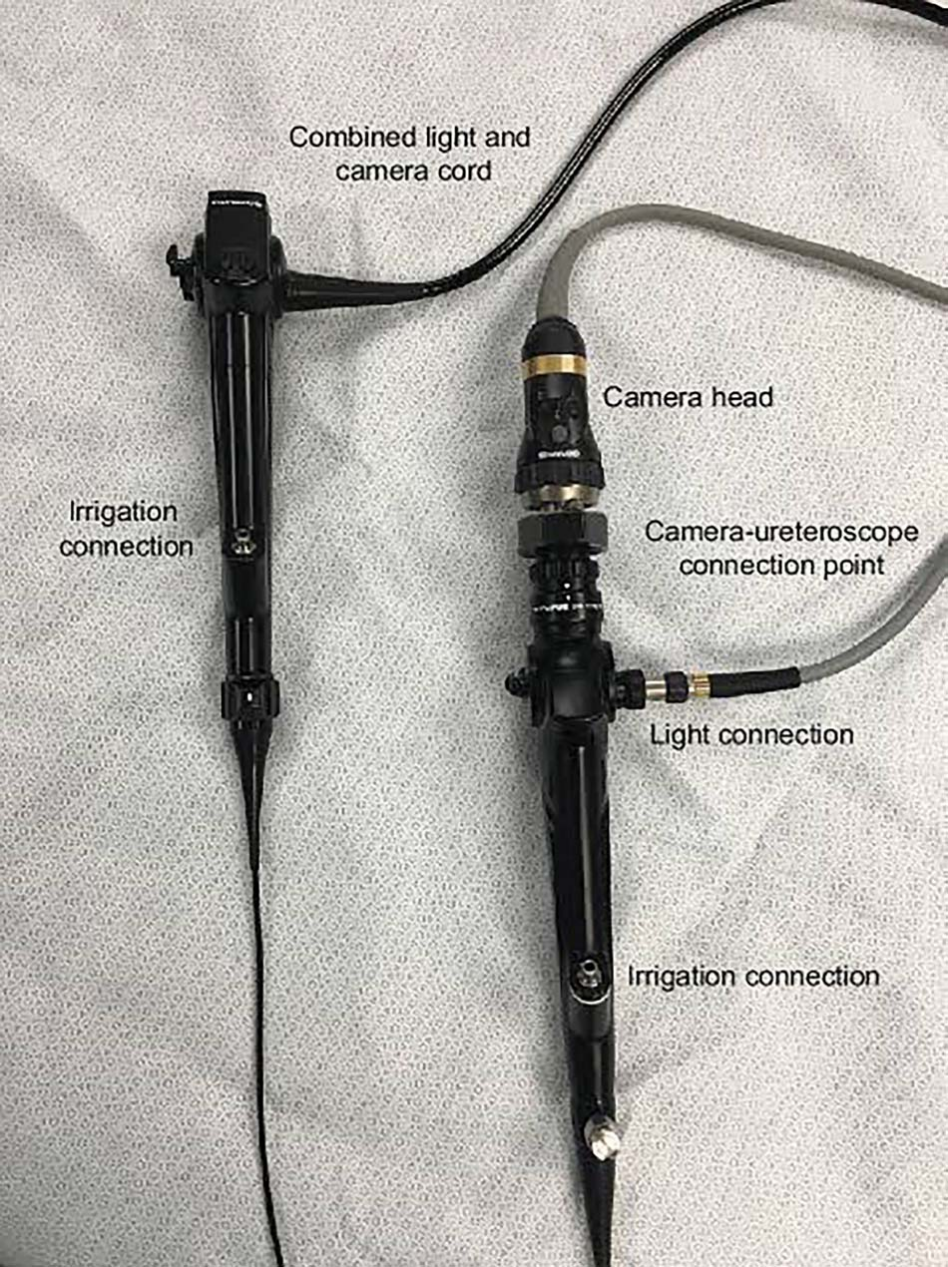
Ureteroscope.

**Figure 2:**
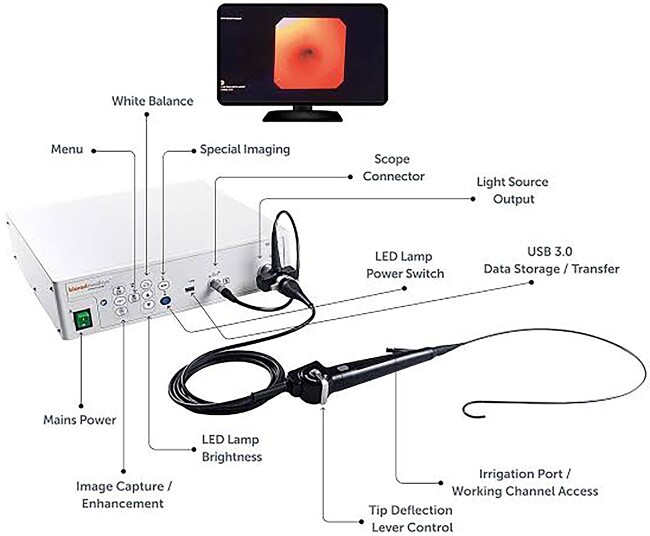
Labelled depiction of ureteroscope with different parts.

Micrognathia, glossoptosis and upper airway obstruction are the three characteristics of the Pierre Robin Syndrome (PRS), a congenital defect that usually coexists with cleft palate [1]. Because one problem triggers a series of events that lead to further indications and symptoms, PRS is not regarded as a syndrome in and of itself but rather as a series of conditions. In the first trimester, mandibular hypoplasia, which can have an underlying condition or not, reduces the oropharyngeal space and causes the tongue to move upward and posteriorly, impairing the ability of the palate to close and obstructing the hypopharynx [1, 2]. We present a case of a PRS-related anatomical anomaly that hindered attempts to manage the airway and the final approach that made it possible to insert an endotracheal tube (ETT). We describe the use of a flexible fiberoptic video-ureteroscope, (Figs 1, 2) as an alternative to a bronchoscope, for airway management. To our knowledge, there is no documentary evidence of the use of a video ureteroscope as an airway endoscope for difficult airway management in a neonate.

## CASE PRESENTATION

A 7-day-old, 2-kg boy was referred to our tertiary care hospital with diagnosed PRS. He had agnathia, glossoptosis and cleft palate. The patient was a preterm child born by normal delivery at 34 weeks of gestation to a middle-class family of south Asian ethnicity. Upon admission, he developed cyanosis with an arterial saturation measured by pulse oximetry (SpO2) of 90% and a venous carbon dioxide pressure (PvCO_2_) of 77.8 mmHg. He had a 0.2-cm mouth opening and hence laryngoscopy was not possible. His saturation was improved to 95% by oxygen with a facial mask. The same night he deteriorated to an SpO2 of 80%. Next, we tried blind nasal intubation with an ETT loaded with stylet, which failed. Then, we tried Size 1 laryngeal mask airway (Well Lead Medical, China) which failed as well. His respiratory distress was improved after we placed an NPA and saturation returned to 100%.

He was planned for correction of the mandible with mandibular distraction osteogenesis (MDO) under general anaesthesia. After giving oxymetazoline nasal drops to dry his secretion, we slowed dialled sevoflurane to 6% and then back to 3% to maintain his spontaneous breathing. Fibreoptic scope (Olympus, Japan) revealed the epiglottis lying on the posterior pharynx, which could not be manoeuvred. Due to repeated attempts, the patient developed laryngospasm, and his (SpO2) was reduced to 70%. Anaesthetic depth was increased with sevoflurane and SpO2 improved to 94%. Once laryngospasm settled, a 2.8-mm outer diameter video ureteroscope was introduced through the nasal cavity. Glottis was visualized but the ETT could not be negotiated into the trachea. Following jaw thrust and slight pulling of the tongue with Magill’s Forceps, a 150-cm long and 0.035-inch diameter atraumatic, Roadrunner® hydrophilic polyurethane-coated (PC) guidewire was introduced through the working channel of the video ureteroscope into the trachea under the vision (Fig. 1) and a 3.5-mm ID ETT was railroaded over it and a definitive airway was established. Once the airway was secured, the patient had an MDO procedure and no adverse events were encountered. He was sent back to neonatal ICU and successfully extubated there on post-operative day 5. Presently, the child is doing fine and only requires minimal oxygen support with nasal prongs.

## DISCUSSION

Infants with PRS may exhibit stridor, chest retraction, sleep apnoea, cyanosis and respiratory insufficiency along with different degrees of upper airway blockage [3, 4]. Furthermore, 25–45% of new-borns with PRS exhibit feeding and swallowing issues, which may be connected to respiratory conditions [4]. During laryngoscopy and intubation, the Pierre Robin Syndrome presents significant difficulty for anaesthesiologists, making oxygenation and ventilation challenging. The airway structure of PRS new-borns makes them one of the most challenging cases to intubate. The anaesthesiologist uses a variety of tools, including the Ambuscope, fibreoptic bronchoscope (FOB) and video laryngoscopes, in difficult airway management situations.

We describe the novel use of a video ureteroscope (Olympus URF-V2) as an airway endoscope. In urology, kidney stones are typically removed with a video ureteroscope. It has a wider field of vision and is longer than a FOB. Currently, sizes with an outside diameter of only 1.8 mm are accessible. In contrast to a FOB, which only offers an angulation of 160° up and 90°, it offers 275° of up- and downangulation [5]. There is little research on video ureteroscopes for airway endoscopy, and there are few sporadic case reports [6]. With a distal tip diameter ranging from 5.3 to 8.7 Fr, flexible ureteroscopes are currently offered in a variety of sizes [5]. We used a 2.8-mm (8.4 Fr) video ureteroscope with a working channel of 1.2 mm to pass the atraumatic, Roadrunner® hydrophilic PC guidewire into the trachea. These guidewires used through a working channel of the ureteroscope are coated with a hydrophilic polymer like polytetrafluoroethylene and have a distal atraumatic tip useful for negotiating difficult ureter [7]. A flexible fibreoptic ureteroscope may be useful in the management of a difficult airway and may become an important tool in the armoury of an anaesthesiologist. At our institute, which is a tertiary care centre, we are now training and utilising video-ureteroscope as an airway endoscope. We have already made use of this vital instrument in several other cases like removal of foreign body, difficult airway management in severe temporomandibular joint ankylosis and also in some cases of one lung ventilation for checking the correct placement of Double lumen tube. Through this case report, we want to highlight the importance of this novel method of airway management which can go a long way in developing new strategies for difficult airway management in the times to come. The legal designate consent has been taken from the father of the child.

## CONCLUSION

We conclude that a flexible fibreoptic ureteroscope may be useful in the management of a difficult airway and may become an important tool for difficult airway management.

## AUTHORS’ CONTRIBUTIONS

A.B. was responsible for the conception and design of the work; R.G. did the analysis of review of literature; S.T. drafted the work and compared our analysis with previous literature on the case. M.K. was responsible for substantively revising the case report. The manuscript has been read and approved by the authors, the requirements for authorship as stated earlier in this document have been met and the author believes that the manuscript represents honest work if that information is not provided in another form. The certificate to this effect will be attached.

## DECLARATION

Data sharing is not applicable to this article as no datasets were generated or analysed during the current study.
